# Effect of non-thermal radiofrequency on body temperature in mice

**DOI:** 10.1038/s41598-020-62789-z

**Published:** 2020-03-31

**Authors:** Thi Cuc Mai, Stéphane Delanaud, Véronique Bach, Anne Braun, Amandine Pelletier, René de Seze

**Affiliations:** 1 INERIS, Experimental Toxicology Unit, National Institute of Industrial Environment and Risks, Parc technologique Alata, Verneuil-en-Halatte, France; 2 PériTox Laboratory, Périnatalité & Risques Toxiques, UMR-I 01 INERIS, Picardie Jules Verne University, Amiens, France

**Keywords:** Physiology, Environmental sciences

## Abstract

Communication technologies based on radiofrequency (RF) propagation bring great benefits to our daily life. However, their rapid expansion raises concerns about possible impacts on public health. At intensity levels below the threshold to produce thermal effects, RF exposure has also recently been reported to elicit biological effects, resembling reactions to cold. The objective of the present study was to investigate the effects of non-thermal RF on body temperature in mice and the related mechanisms. 3-months-old C57BL/6 J mice were exposed to a continuous RF signal at 900 MHz, 20 ± 5 V.m^−1^ for 7 consecutive days, twice per day during the light phase, for one hour each time. The SAR was 0.16 ± 0.10 W.kg^−1^. We showed that body temperature patterns in mice change synchronously with the RF exposure periods. Average body temperature in the light phase in the exposed group was higher than in the control group. The expression of the TRPM8 gene was not affected by RF in trigeminal ganglia. Furthermore, the injection of a TRPM8 antagonist did not induce a temperature decrease in exposed mice, as this was the case for sham-controls. These findings indicate that 900 MHz RF exposure at non-thermal level produce a physiological effect on body temperature in mice. However, the involvement of TRPM8 receptors in the mechanism by which RF induced changes in body temperature of mice which remains to be further explored. It must then be assessed if this effect is extrapolable to man, and if this could lead to consequences on health.

## Introduction

Radiofrequency (RF) is known to carry insufficient quantum energy to eject electrons from atoms, in contrast to energy of CT scans or X-rays in medical applications. However, RF energy can penetrate the body and induce an elevation of temperature in body tissues as used with the microwave oven technology that is referred to as thermal effects of RF. To protect humans from harmful effects induced by excessive tissue heating, safety limits of RF exposure were established and were described in regulatory guidelines, such as those from ICNIRP (International Commission on Non-Ionizing Radiation Protection) and IEEE (Institute of Electrical and Electronics Engineers)^1^.

Up to now, except for the microwave auditory effect^2^, the effects of RF at non-thermal levels remain unexplained, poorly reproducible and controversial. In recent years, studies revealed responses induced in rodents by chronic or repeated RF exposure at low intensity which were similar to those observed in cold conditions. Pelletier *et al*. demonstrated that rats repeatedly exposed (5 weeks, 23 hours per day) to RF at 900 MHz with an intensity of 1 V.m^−1^ at warm ambient temperature, had a subcutaneous tail temperature 1.2 °C lower than controls, due to maintained vasoconstriction^3^. This cannot be explained by a classical dielectric absorption. Also, exposed rats ate more than controls and spent more time to sleep at an ambient temperature of 31 °C, whereas controls spent more time at 28 °C^4^. These results led the authors to suggest that RF exposure induced energy-saving processes. Similarly, Arendash *et al*. showed that repeated RF exposure (twice one hour per day) at 918 MHz with an intensity between 17 and 35 V.m^−1^ induced a body temperature increase of 1 °C in mice after one week of RF exposure, but not in the first days^5,6^.

On the other hand, it has previously been suggested that the thermally sensitive group of the transient receptor potential (TRP) channels could be suitable sensitive targets for mediating RF non-thermal effects^7^. The previously observed responses induced by RF exposure resembling to reactions to cold^3–6^, prompted us to hypothesize that changes in these responses could be a result of the interaction between RF and the TRPM8 receptor, the primary cold sensor in mammals^8^.

TRPM8 behaves as a polymodal receptor activated by cold, pharmacological agents (e.g., menthol and eucalyptol) and chemicals (e.g., icilin and carboxamides) that evoke a cooling sensation^9^. TRPM8 form homotetramers which are abundantly expressed in a subpopulation of primary sensory neurons within the dorsal root and trigeminal ganglia^10^. Biophysical studies show that cold temperatures activate TRPM8 in sensory neurons expressing the channel, causing depolarization through raised concentrations of intracellular sodium and calcium, resulting in transmitting the cold signals to the central thermoregulation^11–13^. *In vivo*, various assays on rodents confirmed the critical role of TRPM8 receptors in the thermoregulatory system. These receptors have been implicated not only in autonomic responses but also in behavioural responses. Pharmacological blocking of TRPM8 channels inhibited the autonomic responses of mice such as shivering, non-shivering thermogenesis and vasoconstriction in reaction to cold below 21 °C^14^. Furthermore, TRPM8 null mice were significantly blunted in behavioural cold defences^8,15,16^.

The objective of the present study was to investigate the effects of a non-thermal radiofrequency exposure on body temperature in mice. And then to explore the involvement of TRPM8 receptors in the mechanism by which RF induced changes in body temperature of mice.

## Results

### Effect of RF exposure on body weight

**Figure 1:**
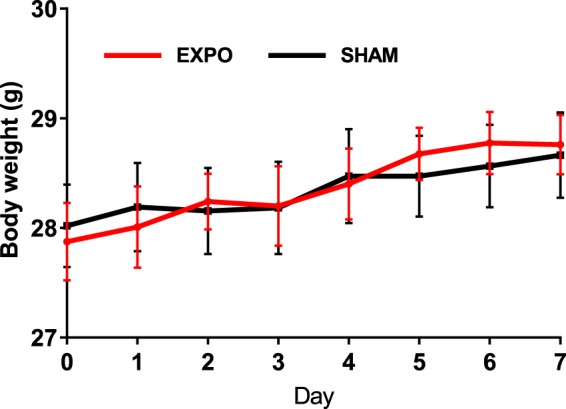
Body weight during the whole exposure of SHAM group (black line, n = 11) and EXPO group (red line, n = 12). The values are shown as means ± s.e.m.

Body weight did not differ significantly between EXPO and SHAM groups during the 7 days of RF exposure (two-way ANOVA, F_1,168_ = 0.02; p = 0.88; n = 12 mice in EXPO group, n = 11 mice in SHAM group) (Fig. 1). This suggests that body weight of mice was not affected by RF exposure.

### Effect of RF exposure on body temperature

In this study, to investigate the effects of RF on body temperature (Temp), mice were exposed twice per day during the light phase, for one hour each time, during 7 days to a continuous RF signal at 900 MHz with an intensity of 20 ± 5 V.m^−1^ (RF “ON” from 9:00 to 10:00 am and from 4:00 to 5:00 pm). Temp data were obtained every 5 min from freely-moving, conscious animals previously implanted with transmitters allowing efficient detection of thermal variability in real time. We checked that transmitters were not affected by RF exposure up to 50 V.m^−1^. Temp data from the exposed group (EXPO) were compared with those of the sham-exposed one (SHAM).

**Figure 2:**
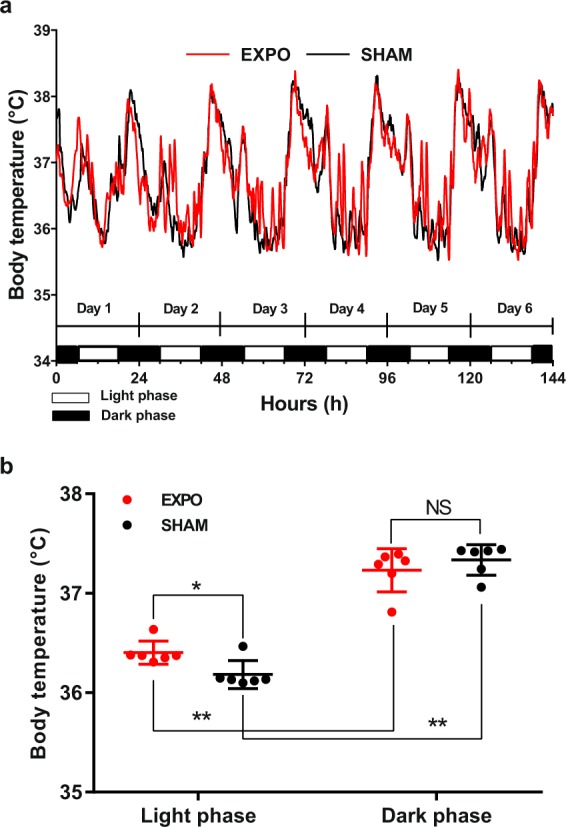
Body temperature from Day 1 to Day 6 of the experiment. (**a**) Body temperature was recorded every 5 min, the values are expressed as mean (n = 11–12). (**b**) Average of body temperature in light phases (from 6:00 am to 6:00 pm) and dark phases (from 6:00 pm to 6:00 am) in 6 days. The values are shown as means ± s.e.m. *p < 0.05, **p < 0.001, Welch’s t test.

Both groups exhibited a strong circadian rhythm with a marked increase at the onset of the dark phase (active period), and a lower Temp during the light phase (rest period) (Fig. 2a). Over the first 6 days, the average variation of Temp between the light and the dark period was 0.9 °C in the EXPO group and 1.3 °C in the SHAM group. In the dark phase, there was no significant difference in the average Temp between the EXPO group and the SHAM group; in contrast, in the light phase, the average Temp in the EXPO group was higher than in the SHAM group (0.23 ± 0.07 °C, p = 0.015) (Fig. 2b).

**Figure 3:**
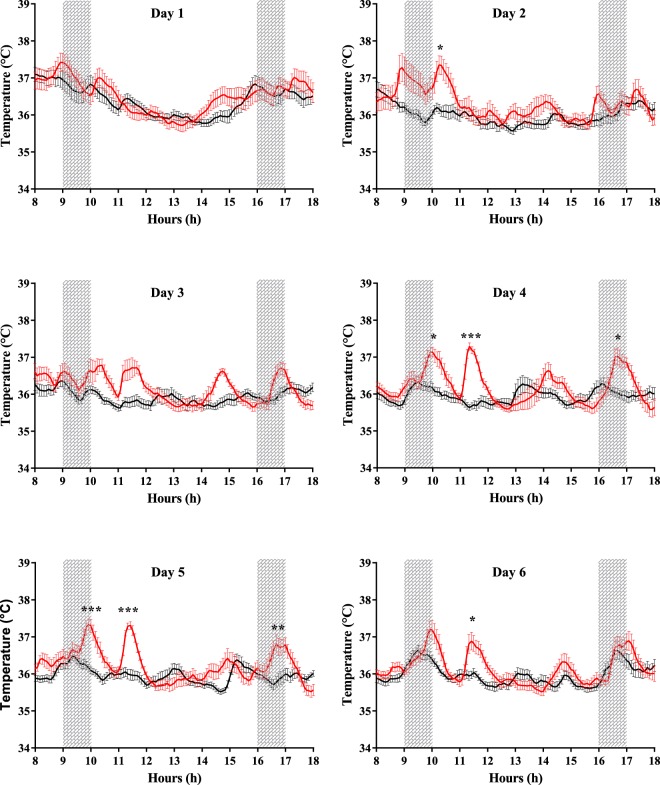
Daily body temperature during the light phase (from 6:00 am to 6:00 pm). Black line indicates SHAM group (n = 11) and red line indicates EXPO group (n = 12). The values are expressed as mean ± s.e.m. Grey areas indicate the period of RF exposure for EXPO group (from 9:00 to 10:00 am and from 4:00 to 5:00 pm). *p < 0.05, **p < 0.01, ***p < 0.001, two-way ANOVA.

We then analysed the daily body temperature in the EXPO and the SHAM groups (Fig. 3). There was no significant difference between both groups on the first day of RF exposure (F_1,7488_ = 0.5, p = 0.46). However, from the second day of the experiment, there were significant differences in body temperature in the exposed group compared to the sham one (F_1,7488_ > 6.0, p < 0.05). In fact, body temperatures of the EXPO group exhibited significant rises in the light phase (rest period), whereas body temperatures in the SHAM group remained around 36 °C (Fig. 3). On the third day of exposure, during the light phase, multiple Temp rises were observed in the EXPO group, but differences did not reach statistical significance. From the fourth to the sixth day of RF exposure, Temp profiles of the EXPO group in light phase exhibited each day similar significant rises over 37 °C at several time points during the two sessions of RF exposure (Fig. 3), whereas Temp of the SHAM group remained around 36 °C (p < 0.05).

Since the duration of each exposure session was one hour, we also analysed the average Temp over one hour during RF exposure and one hour immediately before and immediately after RF exposure, in the morning and in the afternoon. An analysis of variance was used to also test the Day factor. For one-hour periods around the exposure session, there was no significant difference between the SHAM and the EXPO groups depending on the day of the experiment (F_5,936_ = 1.04, p = 0.36). The most interesting result was, whatever the period of exposure during the day, morning or afternoon (F_2,936_ = 3.80, p = 0.02), an increase of the one-hour average of Temp in the EXPO group during (+0.31 °C, p < 0.0001) and after the RF exposure (+0.32 °C, p < 0.0001) whereas there was no significant difference during the hour before the RF exposure. Moreover, the increase of Temp was more important in the morning exposure compared to that of the afternoon exposure (F_1,936_ = 12.18, p = 0.0005; +0.37 °C, p < 0.0001 and +0.13 °C, p = 0.012, respectively).

Surprisingly, after the end of the morning exposure from the fourth to the sixth day of RF exposure, Temp in the EXPO group decreased back to about 36 °C and significantly increased by 1 °C again around 11:30 am.

### Involvement of TRPM8 receptors in the regulation of the body temperature

**Figure 4:**
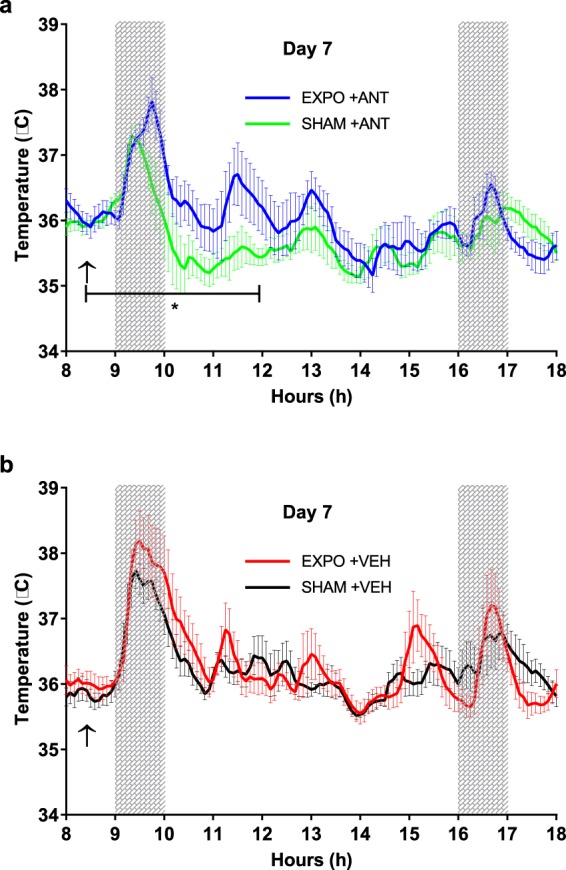
Body temperature on the day 7, after the injection of the TRPM8 antagonist AMG2850 (**a**) and of the vehicle (**b**). The values are expressed as mean ± s.e.m. Arrow indicates the time of AMG2850 injection. Grey areas indicate the period of RF exposure for the EXPO groups. *p < 0.05, two-tailed Mann-Whitney U-tests.

To block the ion channel function of TRPM8, we used a selective TRPM8 antagonist (AMG2850) before the onset of the RF exposure in the morning of the seventh day of the experiment, thus defining new sub-groups: EXPO and SHAM with antagonist = EXPO-ANT and SHAM-ANT; and control EXPO and SHAM with vehicle only = EXPO-VEH and SHAM-VEH. Independently from RF exposure or from the antagonist function, we found that the intra-peritoneal injection induced an increase of about 2 °C after 60 min of the injection (Fig. 4). This phenomenon has been reported as stress from the injection in previous studies^17,18^. Quickly after and for some hours following the AMG2850 injection, SHAM-ANT group showed a significant decrease in Temp of 0.6 °C compared to the other groups during the period from 8:00 to 12:00 am (p < 0.0001). There was no difference in Temp between EXPO-ANT group and EXPO-VEH group which received AMG2850 and the vehicle, respectively.

### Effect of the RF exposure on the expression of TRPM8 mRNA in the trigeminal ganglia

**Figure 5:**
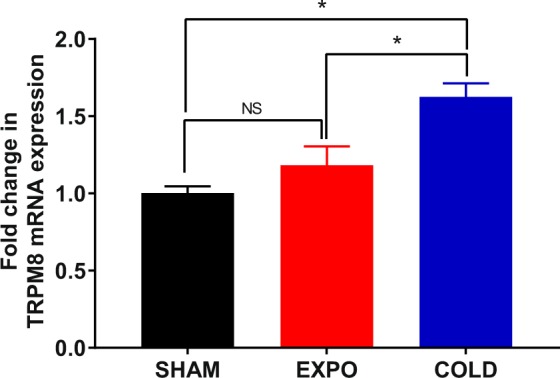
Effect of RF exposure on the expression of TRPM8 mRNA in the trigeminal ganglia. GAPDH and 18 S RNA genes were used as house-keeping genes. Data are expressed as mean ± s.e.m (n = 4 mice). *p < 0.05, two-tailed Mann-Whitney U tests.

To further investigate a possible implication of TRPM8 receptors in RF induced changes in body temperature, we quantified the expression of the TRPM8 gene in the trigeminal ganglia in exposed and sham groups and included an additional positive control group of animals housed in a chamber at an ambient temperature of 5 °C for 7 days without RF exposure (COLD group). As expected, the level of TRPM8 mRNA in the COLD group was significantly upregulated about 1.6 fold relative to the SHAM-VEH and EXPO-VEH groups (p < 0.05) (Fig. 5)^19^. However, no significant difference was found in TRPM8 expression in the trigeminal ganglia between the exposed and the sham groups.

## Discussion

In the present study, we investigated the effects of 900 MHz RF at an intensity of 20 ± 5 V.m^−1^ for twice one hour per day for 7 consecutive days in C57BL/6 J male mice. The SAR was 0.16 ± 0.1 W.kg^−1^.

The body weight of mice was not affected by the RF exposure for 7 days. These findings were similar to other previous short-term studies with higher SAR levels. In Maskey’s study, body weight of mice did not change significantly after RF exposure at 835 MHz with SAR of 1.6 W.kg^−1^ or 4 W.kg^−1^ for 5 days^20^. Zhang *et al*. reported a similar result with mice exposed to 1.8 GHz RF for 4 weeks at a SAR of 2.2 W.kg^−1 ^^21^.

Our data clearly demonstrated that exposure to 900 MHz RF at a SAR of 0.16 W.kg^−1^ significantly affected the body temperature of mice. In our study, the modification of body temperature observed during the constraint-free procedure (Anipill loggers) was only associated with the RF exposure. Moreover, this procedure was efficient to detect thermal variability in real time (every 5 min) and it was not affected by RF exposure into the chamber. Few studies have examined the effects of RF exposure on the body temperature profile in mice and rats using rectal probes (Arendash’s group^6^ or NTP studies^22^). However, the assessment of body temperature by rectal probes could be biased by directly induced stress heating due to the handling.

In our results, the very striking temporal correlation between the end of the temperature rise and the end of the morning exposure session in the EXPO group clearly showed a causal relationship between RF exposure and thermal regulation. Moreover, the 1 °C-increase in body temperature of exposed animals at the end of the morning RF exposure was similar in Arendash’s study^6^. However, the changes in body temperature did not happen on the first day of RF exposure, indicating that there was not a heating by dielectric absorption, but rather a progressive physiological change. Surprisingly, after the end of the morning exposure from day 4 to day 6, body temperature in the EXPO group decreased back to about 36 °C and significantly increased by 1 °C again around 11:30 am. This suggests that the RF-induced process exhibited a rhythmic pattern, the physiological mechanisms of which have to be explored.

The hypothesis of this study was that changes in body temperature could be a result of the interaction between RF and TRPM8 receptors, the primary cold sensor in mammals. SHAM-ANT group showed a significant decrease in body temperature of 0.6 °C compared to the SHAM-VEH group. Such an effect did not happen in exposed mice: there was no difference in body temperature between EXPO-ANT group and EXPO-VEH group which received AMG2850 and the vehicle, respectively. This suggests that TRPM8 receptors have not been inactivated by AMG2850, maybe by a change in their conformation after 6 days of RF exposure. A change of conformation of TRPM8 receptors has been mentioned in previous studies in which TRPM8 can exist in a state called “desensitized state”, after a long exposure to agonists or a continuous stimulation^23^. To further investigate a possible implication of TRPM8 receptors in RF induced changes in body temperature, we studied the expression of the TRPM8 mRNA. However, no significant difference was found in TRPM8 mRNA expression in the trigeminal ganglia between the exposed and the sham groups.

Together, these results suggest that exposure to RF did not impact the TRPM8 mRNA expression levels, but RF may change the state of TRPM8 channels. To get a better understanding on the role of this receptor, studies on TRPM8 in KO mice could be performed. Thermal regulation involves different organs at the peripheral and the central level, and the interaction between low-level RF and thermoregulation might therefore involve many other mechanisms, such as: an activation of other cold receptors (TRPA1); an inhibition of heat receptors (TRPV1, TRPV4); central interaction with the hypothalamus; global interaction with the autonomous nervous system; the modulation of the thyroid hormones function; and/or the intermittent stimulation of thermogenesis through the brown adipose tissue.

To conclude, our data clearly demonstrated that a repeated exposure to 900 MHz radiofrequency at non-thermal level significantly affected the body temperature of mice. The observed changes remained within the physiological amplitude of body temperature oscillations, and therefore did not impair mice health in our study. It is currently not possible to extrapolate whether these temperature changes happen in other species and/or in human. In fact, thermal receptors in humans could be activated in a similar way as in mice. However, underlying mechanisms should be further explored. For example, if these effects are mediated by brown adipose tissue, there is much less brown adipose tissue in human than in rodents, then potential physiological consequences in humans would likely be reduced. Important further steps will need to address underlying mechanisms or modes of interaction between RF and thermoregulation, as well as potential effects of RF exposure on human body temperature.

## Materials and Methods

### Ethics agreement

Animal procedures have been approved by the nationally accredited Regional Directorate for Health, Animal Protection and the Environment (Amiens, France) and the French Ministry of Research with the permit number: 12614. Animals have been treated in accordance with the European guidelines (2010/63/EU) and the French governmental decree 2013-118 on the care and use of laboratory animals.

### Animals housing

3-months-old C57BL/6 J mice have been purchased from Janvier Lab (Le Genest Saint Isle, France). On arrival to the facility, the mice have been housed for one week for acclimation. The animals were housed two per polycarbonate cage (1264 C, Eurostandard Type II 267 ×207 ×140 mm) with stainless steel grids.

Environmental conditions were controlled: 12 hours dark–12 hours light cycle, lights on at 6:00 am and off at 6:00 pm, ambient temperature of 24 ± 1 °C, relative air humidity between 45 and 55%. Food and tap water were available *ad libitum*.

### Exposure system

A whole-body exposure system was designed inside a climatic chamber for *in vivo* experiments to 900 MHz CW radiation. A detailed description of the RF exposure system has previously been published^4^. Briefly, a 900 MHz signal was generated from a radiofrequency power source type RFS7001800–6×0.5 (RFPA, Artigues-près-Bordeaux, France), located outside the climatic chambers and connected to a four-output divider which simultaneously supplied four antennas Kathrein 800–10465 (Rosenheim, Germany). These antennas were set 80 cm above the mice cages and distance between two antennas was 45 cm. In this study, the climatic chamber could accommodate 7 mice cages. To obtain an electromagnetic field as homogeneous as possible for each cage, output power of the four antennas was adjusted to respectively 10 mW, 401 mW, 719 mW and 0 mW. Electric field was measured at 20 ± 5 V.m^−1^ by EP600 Electric field Probe (Narda Safety Test Solutions Srl, Italy) before and during RF exposure in the climatic chamber in the presence of mice. Sham mice were housed in a separate climatic chamber, with identical ambient parameters as in the RF exposure chamber and cages arranged in the same pattern without exposure to RF field signals.

An experimental dosimetry has been performed in the climatic chambers. A mouse phantom was prepared from a gel which exhibits dielectric parameters close to those of the subject’s (mouse) physiological conditions. The composition of the gel phantom includes: 50% (v/v) acrylamid/bis-acrylamid (30% solution, A3574, Sigma-Aldrich), 0.06% (v/v) TEMED (T9281, Sigma-Aldrich), 0.1% (w/v) sodium chloride (Fisher Chemical), 0.06% (w/v) ammonium persulphate (A3678, Sigma-Aldrich) and water^24,25^. The specific heat capacity of the gel was measured using a Calvet calorimeter (C80, Setaram, France).

**Figure 6:**
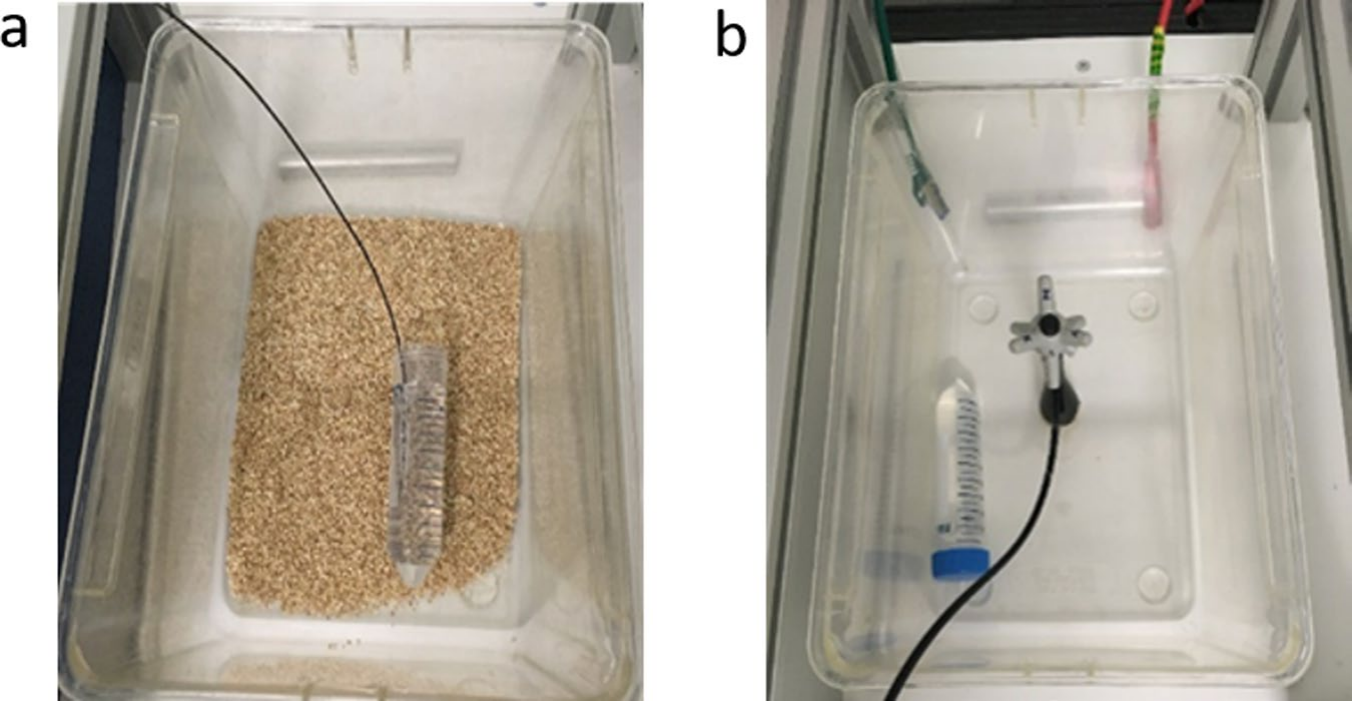
Experimental set up. (**a**) Phantom with a Luxtron temperature probe. (**b**) EP600 Electric probe.

Before the experiments began, one fiber optic temperature sensor (Luxtron) had been inserted into the phantom at a position similar to the peritoneal cavity to monitor the temperature every second. The phantom was allowed to reach an equilibrium with ambient conditions for some hours. 8 measurements were performed, the duration of each session was one hour. The position of the phantom was changed in different cages between measurements. The electric field intensity was set close to the maximal reachable value, 140 V.m^−1^, and below the saturation value which can be measured by an EP600 Electric probe (Fig. 6).

Then the SAR of each measurement was determined from: SAR = c_gel_ ∆T /∆t^25,26^

where c_gel_ is the specific heat capacity of the gel (4090 J.Kg^−1^°C); ∆T/∆t is the initial temperature slope due to the RF heating.

This experiment allowed calculating a SAR value of 7.6 ± 4.9 W.kg^−1^ at the intensity of 140 V.m^−1^. Therefore, SAR at the intensity of 20 V.m^−1^ was determined 0.16 ± 0.10 W.kg^−1^.

### Anipill transmitter implantation

Body temperature of mice was recorded by using Anipill loggers (1.7 g weight and 8.2 mm diameter, 17.2 mm length) (BodyCap, Paris, France) to obtain temperature data from freely-moving mice. The loggers alone were not affected by the RF into the climatic chamber. For surgery, after acclimation, mice have been anesthetized by isoflurane (Piramal Healthcare, Morpeth, United Kingdom) at a concentration of 4% at 2 l.min^−1^ oxygen flow in the induction chamber. When mice were in a moderately deep plane of anaesthesia, oxygen flow and isoflurane concentration have been adjusted to 1 l.min^−1^ and 2.5% respectively. For pre-operative analgesia, meloxicam (Metacam injectable (5 mg.ml^−1^) Boehringer, Ingelheim, Germany) was administered subcutaneously at a dose of 5 mg.kg^−1^. Fur of the abdominal area was shaved, and skin was disinfected with Vetadine Solution (Vetoquinol, Lure, France) followed by 70% alcohol. An incision of 1 cm was made through the skin. Before abdominal muscles incision, lidocaine (Vétoquinol, Lure, France) was instilled on the abdominal wall. Once the Anipill logger had been inserted, muscle and skin were sewed separately with Prolene 4–0 and Mersilk 4–0 suture (Ethicon, United Kingdom). Animals returned to a clean cage for 4 days of recovery prior to experiments.

### Experimental design

The mice have been randomly divided in 3 groups. SHAM mice (n = 11) were placed in the chamber without exposure to RF field signals. EXPO mice (n = 12) were exposed to a continuous RF signal at 900 MHz, 20 ± 5 V.m^−1^ at a SAR of 0.16 ± 0.10 W.kg^−1^ for 7 consecutive days, twice one hour per day (from 9:00 am to 10:00 am and from 4:00 pm to 5:00 pm). A COLD group (n = 6) was housed in a chamber at an ambient temperature of 5 °C for 7 days without RF exposure, as positive control to evaluate the gene expression of TRPM8 receptors.

Thirty minutes prior to the onset of RF exposure in the morning of the seventh day, a TRPM8 channel antagonist, AMG2850 (Alomone Labs, Jerusalem, Israel), was injected intra-peritoneally at a dose of 30 mg.kg^−1^ in 5 mice of the SHAM group (called SHAM-ANT group) and 6 mice of the EXPO group (called EXPO-ANT group). The drug was dissolved in DMSO at a concentration of 20 mg.ml^−1^ and then diluted to 3 mg.ml^−1^ in saline. Vehicle injection (without AMG2850) was injected in 6 SHAM mice (called SHAM-VEH group) and 6 EXPO mice (called EXPO-VEH group).

To avoid inhomogeneity in RF exposure due to metallic components, 20 min prior to every onset of RF exposure, water bottles with stainless-steel nipples have been taken off and metal grids have been replaced by aerated plastic covers (50% linear apertures for air exchange). Normal housing was recovered 15 min after RF exposure. All these experimental manipulations have been performed on both EXPO and SHAM groups. Body temperature data were recorded every 5 min during 7 consecutive days of the experiment.

### Real-time polymerase chain reaction

Trigeminal ganglia have been dissected from all animals, total RNA was extracted using the RNeasy mini kit (Qiagen, Germany) following manufacturer’s instructions by using a fast tissue homogenizer (Percellys 24, Bertin). Samples have been quantified using a Nanodrop ND-8000 spectrophotometer (Nanodrop Technologies, Wilmington, DE).

Reverse transcription of 400 ng mRNA has been performed with Omniscript Reverse Transcription Kit (Qiagen, Germany). The reaction was incubated at 60 °C for 60 min.

Real-time PCR has been performed with Taqman Advanced Fast MasterMix Kit (Fisher Scientific, France) with Taqman Gene expression assays (FAM) for TRPM8 (Mm01299593m_1, Fisher Scientific, France), GAPDH (Mm99999915_g1, Fisher Scientific, France) and 18 S RNA (Mm03928990_g1, Fisher Scientific, France). PCR assays have been performed in 4 mice per group in 96-well plate using the LightCycler 96 System (Roche, Germany).

All samples were run in duplicate wells and no template controls were included. Amplification was performed for 40 cycles. Following an initial hot start of 120 s at 95 °C, each cycle consisted of 3 s of denaturation at 95 °C, and 60 s of annealing at 60 °C. The change of mRNA expression levels of TRPM8 target genes have been normalized to GAPDH and 18 S RNA as the reference genes. The relative quantification of real time RT-PCR products was performed using the equation^27^:

$${\rm{Relative}}\,{\rm{gene}}\,{\rm{expression}}=({({{\rm{E}}}_{{\rm{TRPM8}}})}^{\Delta {\rm{Ct}}{\rm{TRPM8}}})/{\rm{Geomean}}({({{\rm{E}}}_{{\rm{reference}}{\rm{genes}}})}^{\Delta {\rm{Ct}}{\rm{reference}}{\rm{genes}}})$$

### Data analysis

Statistical analyses have been performed using GraphPad Prism version 7.04 for Windows (GraphPad Software, San Diego California USA). The body weight has been analysed by a two-way ANOVA with the Day and the RF exposure as factors. Data on daily body temperature have been analysed by a two-way ANOVA with the Time of day (every 5 min/24 h) and the RF exposure as factors. The average body temperatures in light phases and dark phases over 6 days have been analysed by Welch’s t tests. The data of body temperature after antagonist injection (from 8:00 to 12:00 am) and TRPM8 mRNA levels have been analysed by two-tail Mann-Whitney U Tests. For all tests, P <  0.05 was considered statistically significant.
